# Association entre dépression et charge virale chez les personnes sous traitement antirétroviral suivies à l´Hôpital Central de Yaoundé au Cameroun

**DOI:** 10.11604/pamj.2022.41.320.33056

**Published:** 2022-04-20

**Authors:** Esther Voundi Voundi, Ginette Claude Mireille Kalla, Joel Duplexe Tschoufack Kenfack, Jean Pierre Kamga Olen, Marie Josée Essi, Francois-Xavier Mbopi-Keou

**Affiliations:** 1 Faculté de Médecine et des Sciences Biomédicales, Université de Yaoundé I, Yaoundé, Cameroun,; 2 Institut pour le Développement de l´Afrique (The-IDA), Yaoundé, Cameroun

**Keywords:** Virus de l’immuno déficience humaine, dépression, charge virale, Yaoundé, Cameroun, Human immunodeficiency virus, depression, viral load, Yaounde, Cameroon

## Abstract

**Introduction:**

la dépression pourrait être associée à une réponse immunitaire et virologique médiocre, à une qualité de vie moins bonne et des coûts plus élevés liés à l´utilisation des soins de santé chez les personnes vivant avec le VIH. L´objectif était de rechercher l´association entre la dépression et la charge virale chez les personnes vivant avec le VIH sous traitement antirétroviral, suivies à l´Hôpital Central de Yaoundé.

**Méthodes:**

une étude transversale et analytique a été menée à l´Hôpital Central de Yaoundé sur 8 mois (novembre 2019 à juillet 2020), chez des personnes vivant avec le VIH ayant le résultat de leur charge virale. Avant de débuter l´étude, le consentement éclairé de chaque participant a été obtenu. Les données sociodémographiques, cliniques, paracliniques et le mode de vie ont été collectées. La dépression a été évaluée selon l´échelle Hospital Anxiety and Depression scale (HAD). Le recrutement était consécutif non probabiliste. L´analyse statistique a été faite à l´aide du logiciel SPSS version 23.0. La valeur p < 0,05 a été considérée comme statistiquement significative.

**Résultats:**

des 205 participants enrôlés, le sexe féminin était le plus représenté (n=153, 74,6%) et la moyenne d´âge était de 46,5 ± 1,8 ans. Tous les participants étaient au stade clinique I du VIH et la majorité présentait une charge virale indétectable (n=164, 80,0%). La dépression certaine était retrouvée chez 4,8% des cas et les personnes vivant avec le VIH ayant les symptômes d´une dépression certaine étaient plus susceptibles d´avoir une charge virale élevée (OR = 14,24 [3,61-56,14]; p = < 0,001).

**Conclusion:**

la dépression pourrait favoriser la survenue d´une charge virale élevée.

## Abstract (en)

**Introduction:**

depression may be associated with poor immune and virological response, poor quality of life and high medical costs in people living with HIV. The purpose of this study is to investigate the association between depression and viral load in people living with HIV on antiretroviral treatment followed at the Yaounde Central Hospital.

**Methods:**

we conducted a cross-sectional study of people living with HIV who had their viral load results at the Central Hospital of Yaounde over 8 months (November 2019 to July 2020). Before starting the study, informed consent was obtained from each participant. Sociodemographic, clinical, paraclinical and lifestyle data were collected. Depression was assessed using the Hospital Anxiety and Depression scale (HAD). Consecutive and non-probability sampling was used. Statistical analysis was performed using SPSS software version 23.0. A p-value < 0.05 was considered statistically significant.

**Results:**

of the 205 participants enrolled, female sex was the most represented (n=153, 74.6%) and the mean age was 46.5 ± 1.8 years. All participants had clinical stage I HIV and most of them had undetectable viral load (n=164, 80.0%). Definite depression was found in 4.8% of cases and people living with HIV with symptoms of definite depression were more likely to have a high viral load (OR = 14.24 [3.61-56.14]; p = <0.001). **Conclusion:** depression could be a leading cause of high viral load.

## Introduction

L´infection par le Virus de l´Immunodéficience Humaine (VIH) constitue un problème majeur de santé publique à l´échelle mondiale [1]. L´Afrique reste la région la plus touchée dans le monde [2]. Selon le rapport du Programme commun des Nations Unies sur le VIH/SIDA en 2017, environ 540 000 personnes vivaient avec le VIH au Cameroun avec une incidence de 1,39% [2]. Des progrès considérables ont été effectués dans la prise en charge des personnes infectées, permettant de réduire la mortalité [3]. Les objectifs 90-90-90 stipulaient selon l´Organisation mondiale de la Santé (OMS) qu´en 2020, 90% des personnes infectées connaitront leur statut, 90% des personnes infectées seront sous traitement antirétroviral (TAR) et 90% auront une charge virale indétectable [4,5], ce qui permettrait de réduire considérablement la survenue d´infections opportunistes. [1]. La non-atteinte de ces objectifs pourrait s´expliquer par la non observance au TAR. Des études menées à Odisha en Inde et à Douala au Cameroun en 2015 révèlent que jusqu´à 60% des patients présentent une mauvaise observance médicamenteuse et qu´elle est considérée comme la cause la plus fréquente d´échec du traitement [6,7]. Plusieurs facteurs sont associés à la non observance au TAR, notamment les troubles psychologiques tels que la dépression qui sont courants chez les personnes infectées par le VIH [8].

Selon l´OMS, la dépression constitue un trouble mental courant, caractérisée par la tristesse, la perte d´intérêt ou de plaisir, des sentiments de culpabilité ou de faible estime de soi, des troubles du sommeil ou de l´appétit, d´une sensation de fatigue et d´un manque de concentration [9]. Une méta-analyse a révélé que la probabilité d´atteindre une bonne adhérence au TAR était plus faible chez les personnes présentant des symptômes dépressifs [10]. La dépression pourrait être associée à une réponse immunitaire et virologique médiocre, à la progression vers le SIDA, à une qualité de vie moins bonne et des coûts plus élevés liés à l´utilisation des soins de santé [5,11-13]. Cependant la relation entre la dépression et la charge virale des patients vivant avec le VIH est peu étudiée dans notre contexte. Il nous a donc paru intéressant rechercher l´association entre la dépression et la charge virale chez les personnes vivant avec le VIH sous TAR, afin de ressortir l´intérêt de l´évaluation de la santé mentale dans cette population vulnérable.

## Méthodes

**Type et lieu de l´étude:** une étude transversale analytique a été menée. L´enrôlement des participants s´est effectué dans le Centre de Traitement Agréé (CTA) du VIH de l’HCY. Ce centre a été choisi pour sa grande file active de 12592 patients dont 11709 en première ligne, 780 en deuxième ligne et 103 en troisième ligne en 2018.

**Durée et période de l´étude:** l´étude s´est déroulée du 30 novembre 2019 au 31 juillet 2020, soit huit mois. La collecte des données s´est faite sur une période de 4 mois (12 février au 10 juin 2020).

**Population d´étude:** étaient inclus dans l´étude tous ceux âgés de 18 ans et plus, séropositifs au VIH sous TAR depuis au moins 6 mois, ayant une charge virale disponible et qui ont accepté de participer à l´étude. Ont été exclus tous ceux n´ayant pas respecté la prise de TAR au cours du mois précédent et les femmes enceintes. L´échantillonnage était consécutif non probabiliste.

**Procédure:** après avoir expliqué l´objet et le but de l´étude, le formulaire de consentement éclairé a été présenté à tous les participants répondant à nos critères d´inclusion. Pour ceux qui ont accepté de participer à l´étude, les variables sociodémographiques, cliniques, paracliniques, ainsi que le mode de vie ont été collectées à l´aide d´une fiche technique préétablie. L´évaluation de la dépression s´est faite selon l´échelle « Hospital Anxiety and Depression scale » (HAD) [14]. Cette dernière comporte 14 items cotés chacun de 0 à 3 dont sept questions se rapportent à la dimension dépressive (total D) permettant ainsi l´obtention d´un score avec une note maximale de 21. L´interprétation de ce score s´est faite de la manière suivante: ≤ 7 = absence de symptomatologie, 8 à 10= symptomatologie douteuse, ≥11= symptomatologie certaine [14]. Un avis spécialisé était requis en cas de symptomatologie douteuse à certaine pour une meilleure prise en charge des participants.

**Variables de l´étude:** les données sociodémographiques (âge, sexe, statut professionnel, niveau d´instruction, statut matrimonial, religion), cliniques (stade clinique OMS de la maladie, la durée du traitement en jours), paracliniques (charge virale) et le mode de vie (utilisation du préservatif au cours des rapports sexuels, consommation de drogues illicites, consommation d´alcool, usage du tabac) ont été collectées à l´aide d´une fiche technique préétablie.

**Analyses statistiques:** les données étaient saisies et analysées avec le logiciel SPSS (Statistical Package for the Social Sciences) version 23.0. Les variables qualitatives étaient exprimées sous forme de fréquences et pourcentages et les variables quantitatives sous forme de moyenne et écart-type. Les proportions ont été comparées à l´aide d´un test de Chi^2^ ou le test exact de Fisher quand cela était indiqué. La force d´association a été estimée par l´Odds ratio et l´intervalle de confiance à 95%. Le niveau de signification statistique a été fixé à une valeur p <0,05. Dans le but d´exclure l´effet des facteurs de confusion, l´analyse multivariée a été faite sur le modèle de la régression logistique, en incluant toutes les variables dont la valeur p était inférieure à 0,05. La variable indépendante étudiée était la dépression certaine.

**Considérations éthiques:** la clairance éthique ainsi que le consentement éclairé des participants ont été obtenus. Les informations collectées étaient exploitées exclusivement dans le cadre de l´étude et l´anonymat conservé.

## Résultats

**Figure 1:**
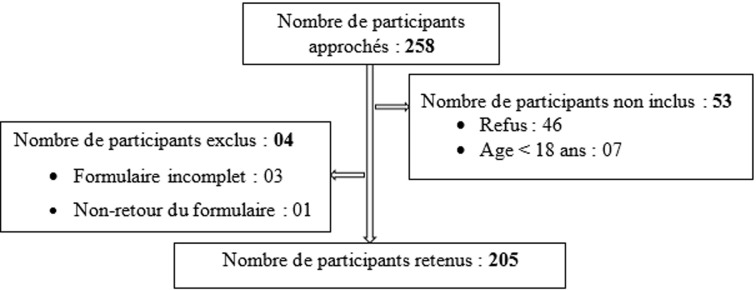
diagramme de déroulement du recrutement

Au total, 205 participants ont été retenus soit un taux de participation de 79,5% (Figure 1).

**Caractéristiques de la population d´étude:** le sexe féminin était majoritairement représenté (n=153; 74,6%), pour un sexe ratio de 2,9. L´âge moyen dans la population d´étude était de 46,5 ± 1,8 ans, avec des extrêmes de 25 et 80 ans. La plupart des participants avait un emploi (n=166; 81,0%). La majorité avait fait des études secondaires (n=119; 58,0%.), suivie de ceux ayant fait des études primaires (n=62; 30,2%). Les participants célibataires étaient les plus représentés (n=112 ; 54,6%). Les participants de religion chrétienne représentaient la quasi-totalité de notre population, soit 200 (97,6%). La fréquence des participants entretenant des rapports sexuels était de 67,3% (n=138), parmi lesquels 45,7% (63/138) utilisaient irrégulièrement le préservatif. Les fréquences de consommation des boissons alcoolisées et de tabac étaient de 56,6% (n=116), et de 3,9% (n=8) respectivement. Tous les participants au cours de l´étude étaient au stade clinique I du VIH (Tableau 1). La majorité des participants avait une charge virale indétectable (n=164; 80,0%), suivie de ceux présentant une charge virale élevée (≥ 1000 copies/ml) (n=25; 12,2%). Les participants qui avaient une durée de consommation des antirétroviraux de plus de 60 mois (5ans), représentaient près des trois quarts de notre population (n=153; 74,6%) (Tableau 2).

**Tableau 1:** caractéristiques sociodémographiques de la population d´étude

Variables	Effectif	Pourcentage (%)
**Sexe (N=205)**		
Masculin	52	25,4
Féminin	153	74,6
**Tranche d´âge (années) (N=205)**		
25-35	32	15,6
35-45	60	29,3
45-55	66	32,2
55-65	37	18
>65	10	4,9
**Profession (N=205)**		
Sans emploi	21	10,2
Employé	166	81,0
Retraité	18	8,8
**Niveau d´étude (N=205)**		
Primaire	62	30,2
Secondaire	119	58
Supérieur	24	11,7
**Statut matrimonial (N=205)**		
En union	93	45,4
Célibataire	112	54,6
**Réligion (N=205)**		
Chrétienne	200	97,6
Musulmane	5	2,4
**Aucune activité sexuelle (N=205)**		
Oui	67	32,7
Non	138	67,3
**Usage du préservatif au cours des rapports sexuels (N=138)**		
Régulièrement	75	54,3
Irrégulièrement	63	45,7
**Consommation de drogues illicites (N=205)**		
Oui	2	1
Non	203	99
**Consommation des boissons alcoolisées (N=205)**		
Oui	116	56,6
Non	89	43,4
**Consommation du tabac (N=205)**		
Oui	8	3,9
Non	197	96,1

**Tableau 2:** caractéristiques clinico-biologiques de la population d´étude

Variables	Effectif N=205	Pourcentage (%)
**Stade clinique OMS**		
Stade I	205	100
**Charge virale (copies/ml)**		
Indétectable <40	164	80,0
40-1000	16	7,8
**≥** 1000	25	12,2
**Durée du traitement (mois)**		
[6-24[	18	8,8
[24-60[	34	16,6
≥ 60	153	74,6

**Evaluation de la dépression (Echelle HAD):** au cours de notre étude, 4,8% (n=10) des participants avaient une dépression certaine, contre 77,6% (n=159) de participants n´ayant pas de symptômes de dépression.

**Analyse bivariée:** concernant les caractéristiques sociodémographiques, la religion chrétienne constituait un facteur protecteur de la dépression certaine (OR= 0,07 [0,01-0,43], p = 0,020)(Tableau 3). Une association a été retrouvée entre la dépression et la charge virale en ce sens que les participants présentant les signes de dépression certaine étaient 14 fois plus susceptibles d´avoir une charge virale élevée (OR= 13,89 [3,60-53,64], p < 0,001) tandis que ceux avec charge virale indétectable avaient 0,51 fois un moindre risque de dépression (OR= 0,51 [0,10-0,25], p < 0,001) (Tableau 3).

**Tableau 3:** facteurs sociodémographiques et clinico-biologiques associés à la dépression certaine de la population d´étude (analyse bivariée)

Variables	Dépression certaine		OR^1^ [IC^2^ 95 %]	p
	Oui N=10 n (%)	Non N=195 n (%)		
**Age (en année)**				
< 45	5 (50,0)	108 (55,4)	0,81 [0,23-2,86]	0,493
≥ 55	3 (30,0)	44 (22,6)	1,47 [0,36-5,88]	0,412
**Sexe**				
**Féminin**	7 (70,0)	146 (74,9)	0,78 [0,19-3,14]	0,487
**Proféssion**				
Employé	7 (70,0)	159 (81,5)	0,53 [0,13-2,14]	0,291
Retraité	2 (20,0)	16 (8,2)	2,78 [0,55-14,30]	0,215
Sans emploi	1 (10,0)	20 (10,30)	0,97 [0,12-8,08]	0,727
**Niveau d´étude**				
Primaire	4 (40,0)	58 (29,7)	1,57 [0,43-5,79]	0,355
**Statut matrimonial**				
En union	3 (30,0)	90 (46,2)	0,50 [0,13-2,00]	0,253
**Réligion**				
Chrétienne	8 (80,0)	192 (98,5)	**0,07 [0,01-0,43]**	**0,020**
**Consommation**				
Boisson alcoolisée	4 (40,0)	112 (57,4)	0,70 [0,32-1,50]	0,223
Tabac	0 (0,0)	8 (4,1)	Indéfini	0,665
Substances illicites	0 (0,0)	2 (1,0)	Indéfini	0,905
**Durée du traitement antirétroviral (en mois)**				
≥ 60	24 (58,5)	129 (78,7)	1,08 [0,78-1,48]	0,513
**Paraclinique: charge virale (copies/ml)**				
< 40	2 (20,0)	162 (83,1)	**0,51 [0,10-0,25]**	**< 0,001**
≥ 1000	6 (60,0)	19 (9,7)	**13,89 [3,60-53,64]**	**< 0,001**
1. OR : Odds Ratio 2. IC: Intervalle de Confiance				

**Analyse multivariée:** après exclusion des facteurs de confusion, une association indépendante et significative a été retrouvée entre la dépression certaine et la charge virale élevée (OR = 14.24 [3.61-56.14]; p = <0.001) (Tableau 4).

**Tableau 4:** facteurs sociodémographiques, cliniques et paracliniques associés à la dépression certaine (analyse multivariée)

Variables	Dépression certaine		OR ajusté [IC95 %]	p ajustée
	Oui N=10 n (%)	Non N=195 n (%)		
**Réligion chrétienne**	8 (80,0)	192 (98,5)	0,335 [0,07-1,58]	0,187
**Charge virale ≥ 1000 copies/ml**	6 (60,0)	19 (9,7)	**14,24 [3,61-56,14]**	**<0,001**

## Discussion

Alors que le continent africain continue de supporter le plus lourd fardeau de l´infection au VIH dans le monde [15], les travaux de recherche portant sur l´épidémiologie des troubles psychologiques tels que la dépression et leur influence sur la charge virale dans les populations infectées par le VIH sont quasi inexistants dans notre contexte. L´objectif général de notre étude était de rechercher l´association entre la dépression et la charge virale chez les personnes vivant avec le VIH sous traitement antirétroviral suivies à l´Hôpital Central de Yaoundé. L´âge moyen dans la population d´étude était de 46,5 ± 1,8 ans, avec des extrêmes de 25 et 80 ans. Ces résultats se rapprochent de ceux obtenus par Mbopi Keou *et al*. en 2012, et par Ba *et al*. en 2018 qui retrouvaient un âge moyen de 41,28 et 44 ans respectivement [16,17]. Il diffère de celui obtenu par Billong *et al*. en 2013 ; et Fokam *et al*. en 2016 qui avaient retrouvé 35 et 37 ans respectivement [18,19]. Ceci pourrait s´expliquer par le fait que la présente étude n´incluait pas les patients jeunes, âgés de moins de 18 ans. La population d´étude était majoritairement représentée par les participants de sexe féminin (74,6%).

Cette féminisation de l´infection au VIH dans nos régions a été reportée par plusieurs auteurs et rapports [16, 19, 20]. La totalité des participants étaient au stade clinique I du VIH. Ce résultat pourrait s´expliquer par le fait que tous les participants de l´étude n´étaient pas enrôlés en contexte d´hospitalisation. La charge virale supérieure ou égale à 1 000 copies/ml était retrouvée chez 12,2% des participants. Ce résultat se rapproche de celui obtenu par Ba *et al*. en 2018 au Sénégal, qui retrouvaient 17% de patients présentant une charge virale supérieure à 1000 copies/ml. Cette prévalence en baisse pourrait traduire une avancée significative au regard des efforts mis en œuvre par les gouvernements durant les cinq dernières années pour l´atteinte du troisième 90 dans les objectifs 90 - 90 - 90. La prévalence de symptômes de dépression certaine de 4,8% a été déterminée en utilisant le score HAD. Il s´agit d´une mesure de dépistage utile pour la présence de symptômes d´anxiété et de dépression [14] qui a été largement appliquée et validée dans différents contextes. Cependant, la comparabilité avec d´autres études ayant évalué les symptômes psychiatriques dans différentes populations utilisant le score HAD sont difficiles en raison des différences méthodologiques entre les études telles que: la conception de l´étude, la population, les critères utilisés pour définir l´anxiété et la dépression, et la disponibilité des données pour chaque niveau de symptômes. Bernard *et al*. en 2017 en Afrique du Sud, ont décrit une prévalence de symptômes de dépression variant entre 14 à 32% chez les PVVIH sous TAR dans une méta analyse [21]. Cette différence pourrait s´expliquer par la difficulté à diagnostiquer les symptômes de dépression, notre étude s´étant principalement focalisée sur la dépression certaine. Il en ressort la nécessité pour chaque PVVIH de consulter un spécialiste au moindre signe de dépression.

Dans notre étude, à l´analyse multivariée, aucun facteur sociodémographique n´a été retrouvé associé à la dépression certaine; cependant, une charge virale élevée était un facteur indépendant significativement associé à une dépression certaine. En effet, une PVVIH en état de dépression avait 14 fois plus de risque d´avoir une charge virale élevée. Par contre, Nyongesa *et al*. au Kenya en 2019 n´ont retrouvé aucune association entre la dépression et la charge virale [22]. Ceci pourrait être dû à la différence dans le mode de recrutement, de la population étudiée et des méthodes d´évaluation de la dépression. Feuillet *et al*. en France ont également décrit une association entre la dépression et la charge virale élevée indépendamment de la mauvaise observance au traitement [23]. Les signes et symptômes retrouvés dans la dépression tels que l´anorexie et l´asthénie pourraient influencer la capacité d´absorption des antirétroviraux par déficit d´apport en nutriments tel que relevé dans certaines études [24]. Parmi les limites de ce travail, il faut noter l´absence d´un médecin référent psychiatre. En effet, le score HAD est une mesure de dépistage utile pour la présence de symptômes d´anxiété et de dépression. Il n´est pas une échelle diagnostique ce qui pourrait poser un problème de classification des sujets. On note par ailleurs le type d´étude choisi, une étude comparative randomisée serait indiquée pour ressortir le lien de causalité.

## Conclusion

La prévalence de la dépression certaine était de 4,8% et elle pourrait être associée à une charge virale élevée chez les PVVIH adhérents au traitement. La probabilité de l´impact nutritionnel lié à la dépression sur l´absorption des antirétroviraux malgré une bonne observance au TAR est à élucider. Il parait important de multiplier, décentraliser et renforcer les actions des structures de dialogue et de soutien psychologique aux personnes vivant avec le VIH. Prendre en charge les troubles de santé mentale des PVVIH devient primordial pour améliorer leur réponse virologique au traitement.

### Etat des connaissances sur le sujet

- Selon l´OMS, la dépression constitue un trouble mental courant, caractérisée par la tristesse, la perte d´intérêt ou de plaisir, des sentiments de culpabilité ou de faible estime de soi, des troubles du sommeil ou de l´appétit, d´une sensation de fatigue et d´un manque de concentration;
- Une méta-analyse a révélé que la probabilité d´atteindre une bonne adhérence au TAR était plus faible chez les personnes présentant des symptômes dépressifs.

### Contribution de notre étude à la connaissance

- La prévalence de la dépression certaine était de 4,8% il existe une association entre l´état de dépression et la charge virale. Il parait important de multiplier, décentraliser et renforcer les actions des structures de dialogue et de soutien psychologique aux personnes vivant avec le VIH.

## Remerciements

L´équipe de recherche adresse de vifs remerciements au staff du CTA de l´Hôpital Central de Yaoundé et à tous les participants à cette étude.
